# Author Correction: New elevation data triple estimates of global vulnerability to sea-level rise and coastal flooding

**DOI:** 10.1038/s41467-019-13552-0

**Published:** 2019-12-12

**Authors:** Scott A. Kulp, Benjamin H. Strauss

**Affiliations:** Climate Central, Palmer Square #402, Princeton, NJ 08542 USA

**Keywords:** Climate-change impacts, Projection and prediction

Correction to: *Nature Communications* 10.1038/s41467-019-12808-z, published online 29 October 2019.

The authors became aware of a mistake in the original version of this Article. Specifically, a minor error was found in the code during peer review that produced small changes in the results. However, the revised numbers were inadvertently omitted from the final version of the manuscript. As a result of this, the following changes have been made to the originally published version of this Article:

The final sentence of the Abstract originally incorrectly read ‘We estimate one billion people now occupy land less than 10 m above current high tide lines, including 250 M below 1 m.’ The correct version states ‘230 M’ in place of ‘250 M’.

The last sentence of the sixth paragraph of the ‘Global’ section of the Results originally incorrectly read ‘This figure doubles the median SRTM-based estimate of 95 million under high emissions and Antarctic instability (RCP 8.5 and K17),…’ The correct version says ‘94 million’ in place of ‘95 million’.

The second sentence of the first paragraph of the ‘National’ section of the Results originally incorrectly said ‘China alone accounts for 15–28% of global ECWL exposure across DEMs,…’ The correct version says ‘18–32%’ in place of ‘15–28%’.

The second sentence of the second paragraph of the same section originally incorrectly read ‘As indicated by CoastalDEM, Bangladesh, India, and Vietnam come to rival China in the median number of people living on land implicated by 2100, totaling 21–30 million even under the low emissions scenario (K14/RCP 2.6), compared to 9–19 M today, and with another 10–25 million on land threatened by annual storm surge.’ The correct version says ‘7–20 million’ in place of ‘10–25 million’.

The first sentence of the fourth paragraph of the same section originally incorrectly read ‘Outside of Asia and excluding the Netherlands, where an extensive flood control network is not captured by any of the elevation models studied, CoastalDEM indicates that 20 other countries are expected to see land currently home to 10% or more of their total populations fall below end-of-century high tide lines (based on median estimates),…’ The correct version says ‘19 other countries’ in place of ‘20 other countries’.

The final sentence of the same paragraph originally incorrectly read ‘Except for Djibouti, Guyana, and the United Arab Emirates, all of these are island nations, and thirteen are classified by the United Nations as Small Island Developing States (SIDS).’ The correct version says ‘Djibouti and Guyana’ in place of ‘Djibouti, Guyana, and the United Arab Emirates’.

The third sentence of the seventh paragraph of the ‘Sensitivity analysis’ section of the Results originally incorrectly said ‘For example, at the 1-degree-error resolution, Bangladesh, India, and Vietnam have CI’s of (−56 to 54%), (−40 to 27%), and (−34 to 23%) about their respective medians, while China is predictably less sensitive at (−21 to 21%).’ The correct version says ‘(−43 to 54%), (−40 to 27%), and (−29 to 23%)’ in place of ‘(−56 to 54%), (−40 to 27%), and (−34 to 23%)’.

Finally, in the original version of Table 1, the last row of the eighth column incorrectly read ‘500 (390–640)’. The correct version replaced this with ‘480 (380–630)’

This has been corrected in both the PDF and HTML versions of the Article. The authors confirm that the above error does not affect the main results or conclusions of the paper. The Supplementary Data were generated automatically during analysis and so were not affected.

