# Supplementary material for: New elevation data triple estimates of global vulnerability to sea-level rise and coastal flooding
Source: Nat Commun. 2019 Oct 29;10:4844. doi: 10.1038/s41467-019-12808-z (PMC6820795; doi:10.1038/s41467-019-12808-z)
Supplement: Supplementary file 2 — Supplementary Information [file 41467_2019_12808_MOESM2_ESM.pdf]

New Elevation Data Triple Estimates of Global Vulnerability to Sea-Level Rise and Coastal Flooding

Supplementary Information

Kulp et al.

Supplementary Table 1

Model	Year	RCP 2.6	RCP 4.5	RCP 8.5
**K14**	2050	0.24 (0.18-0.33)	0.26 (0.18-0.35)	0.29 (0.21-0.39)
	2100	0.49 (0.28-0.84)	0.59 (0.35-0.95)	0.79 (0.51-1.23)
**K17**	2050	0.23 (0.12-0.41)	0.26 (0.14-0.43)	0.31 (0.17-0.48)
	2100	0.56 (0.26-0.98)	0.91 (0.50-1.58)	1.46 (0.93-2.43)

Global mean sea-level rise projections. Models include K14[1], a probabilistic sea-level rise projection that makes simplifying assumptions about Antarctic ice sheet contributions, and K17[2], a non-probabilistic projec-tion that incorporates physical models of ice sheet dynamics. Units are in meters. Median + 90% CI are presented.

Supplementary References

[1] Kopp, R. E. et al. Probabilistic 21st and 22nd century sea-level projections at a global network of tide-gauge sites. Earth's Future 2, 383–406 (2014).

[2] Kopp, R. E. et al. Evolving Understanding of Antarctic Ice-Sheet Physics and Ambiguity in Probabilistic Sea-Level Projections. Earth's Future 5, 1217–1233 (2017).
