# Supplementary material for: New elevation data triple estimates of global vulnerability to sea-level rise and coastal flooding
Source: Nat Commun. 2019 Oct 29;10:4844. doi: 10.1038/s41467-019-12808-z (PMC6820795; doi:10.1038/s41467-019-12808-z)
Supplement: Supplementary file 3 — Description of Additional Supplementary Files [file 41467_2019_12808_MOESM3_ESM.pdf]

Description of Additional Supplementary Files

File Name: Supplementary Data 1

Description: Total current population (millions) occupying vulnerable land. Totals evaluated using CoastalDEM, SRTM, JAXA and MERITDEM elevation datasets, for present-day sea level and under multiple RCP scenarios for 2050 and 2100. Global and country assessments are included, as well as the collective exposure of the small island developing states (SIDS). Permanent exposure (perm) implies elevation below the future local high tide line, while RL1 exposure adds the local 1-year coastal flood return level. Results are presented as 5th, 50th and 95th percentiles based on probability distributions of future sea levels, in the case of projections using K14; and based on simulation frequency distributions, in the case of projections using K17. Locations with maximum exposure less than 0.01M removed.

File Name: Supplementary Data 2

Description: Total current population (millions) occupying vulnerable land by country and globally according to CoastalDEM, based on water heights 0-10 m above local MHHW. Locations with maximum exposure less than 0.01M under both CoastalDEM and SRTM were removed.

File Name: Supplementary Data 3

Description: Total current population (millions) occupying vulnerable land by country and globally according to SRTM, based on water heights 0-10 m above local MHHW. Locations with maximum exposure less than 0.01M under both CoastalDEM and SRTM were removed.

File Name: Supplementary Data 4

Description: Percentage of current population occupying vulnerable land. Percentages evaluated using CoastalDEM, SRTM, JAXA and MERITDEM elevation datasets, for present-day sea level and under multiple RCP scenarios for 2050 and 2100. Global and country assessments are included, as well as the collective exposure of the small island developing states (SIDS). Permanent exposure (perm) implies elevation below the future local high tide line, while RL1 exposure adds the local 1-year coastal flood return level. Results are presented as 5th, 50th and 95th percentiles based on probability distributions of future sea levels, in the case of projections using K14; and based on simulation frequency distributions, in the case of projections using K17. Locations with maximum exposure less than 0.01M removed.

File Name: Supplementary Data 5

Description: Sensitivity analysis. In order to assess the sensitivity of exposure analysis to DEM vertical error, 100 simulated error surfaces are generated at a number of spatial resolutions (from one pixel (1PX, equal to 3 arcseconds) through one degree) and applied to CoastalDEM. The numbers of people (thousands) living less than 2 m above local mean higher high water, according to each of the altered DEMs, are computed in each country and globally, and the 5th, 50th, and 95th percentiles of the resulting distributions are presented. Also presented are total national/global populations, and populations living less than 2 m above MHHW as assessed when using CoastalDEM without any modification.
