# Supplementary material for: New elevation data triple estimates of global vulnerability to sea-level rise and coastal flooding
Source: Nat Commun. 2019 Oct 29;10:4844. doi: 10.1038/s41467-019-12808-z (PMC6820795; doi:10.1038/s41467-019-12808-z)
Supplement: Supplementary file 9 — Reporting Summary [file 41467_2019_12808_MOESM9_ESM.pdf]

nature research

Corresponding author(s): *Double-blind peer review submissions: write DBPR and your manuscript number here instead of author names.*

Last updated by author(s): *YYYY-MM-DD*

Reporting Summary

Nature Research wishes to improve the reproducibility of the work that we publish. This form provides structure for consistency and transparency in reporting. For further information on Nature Research policies, see Authors & Referees and the Editorial Policy Checklist.

Statistics

For all statistical analyses, confirm that the following items are present in the figure legend, table legend, main text, or Methods section.

n/a	Confirmed	
☒	☐	The exact sample size (*n*) for each experimental group/condition, given as a discrete number and unit of measurement
☒	☐	A statement on whether measurements were taken from distinct samples or whether the same sample was measured repeatedly
☒	☐	The statistical test(s) used AND whether they are one- or two-sided<br>*Only common tests should be described solely by name; describe more complex techniques in the Methods section.*
☒	☐	A description of all covariates tested
☐	☒	A description of any assumptions or corrections, such as tests of normality and adjustment for multiple comparisons
☐	☒	A full description of the statistical parameters including central tendency (e.g. means) or other basic estimates (e.g. regression coefficient) AND variation (e.g. standard deviation) or associated estimates of uncertainty (e.g. confidence intervals)
☒	☐	For null hypothesis testing, the test statistic (e.g. *F*, *t*, *r*) with confidence intervals, effect sizes, degrees of freedom and *P* value noted<br>*Give P values as exact values whenever suitable.*
☒	☐	For Bayesian analysis, information on the choice of priors and Markov chain Monte Carlo settings
☒	☐	For hierarchical and complex designs, identification of the appropriate level for tests and full reporting of outcomes
☒	☐	Estimates of effect sizes (e.g. Cohen's *d*, Pearson's *r*), indicating how they were calculated

Our web collection on statistics for biologists contains articles on many of the points above.

Software and code

Policy information about availability of computer code

Data collection	Custom Matlab Code (R2017b) was used to generate CoastalDEM. This code can be made available to editors and reviewers upon request, but due to licensing restrictions from Climate Central, may not be shared publicly.
Data analysis	Custom Python/C++/Matlab (R2017b) code was used to perform exposure analyses. This code can be made available to editors and reviewers upon request, but due to licensing restrictions from Climate Central, may not be shared publicly.

For manuscripts utilizing custom algorithms or software that are central to the research but not yet described in published literature, software must be made available to editors/reviewers. We strongly encourage code deposition in a community repository (e.g. GitHub). See the Nature Research guidelines for submitting code & software for further information.

Data

Policy information about availability of data

All manuscripts must include a data availability statement. This statement should provide the following information, where applicable:

- Accession codes, unique identifiers, or web links for publicly available datasets
- A list of figures that have associated raw data
- A description of any restrictions on data availability

All exposure analyses that support the findings of our study are available within the article and its supplementary information files. The datasets SRTM, AW3D30, MERITDEM, Landscan 2010, and GADM are publically available from their respective owners. The 3-arcsecond (90-meter) version of CoastalDEM used in this analysis is available at no cost from Climate Central for non-commercial research use.

Field-specific reporting

Please select the one below that is the best fit for your research. If you are not sure, read the appropriate sections before making your selection.

☐ Life sciences ☐ Behavioural & social sciences ☒ Ecological, evolutionary & environmental sciences

For a reference copy of the document with all sections, see nature.com/documents/nr-reporting-summary-flat.pdf

Ecological, evolutionary & environmental sciences study design

All studies must disclose on these points even when the disclosure is negative.

Study description	New elevation data was used to assess vulnerability of populated coastal land to sea level rise and coastal flooding. Elevation data were thresholded against projected sea level rise and 1-year storm heights to produce inundation surfaces, and these surfaces were intersected with population density data to estimate exposure.
Research sample	NOAA Coastal Lidar: https://coast.noaa.gov/dataviewer/#/<br>SRTM 3.0 (NASA): https://www2.jpl.nasa.gov/srtm/<br>AW3D30 (JAXA): https://www.eorc.jaxa.jp/ALOS/en/aw3d30/index.htm<br>MERITDEM: http://hydro.iis.u-tokyo.ac.jp/~yamadai/MERIT_DEM/<br>GADM 2.0: https://gadm.org/<br>Landscan 2010: https://landscan.ornl.gov/
Sampling strategy	Not relevant, as the complete datasets listed above were used (no random sampling).
Data collection	SAK collected/downloaded and preprocessed the data to a consistent form suitable for analysis.
Timing and spatial scale	Near-global coastal spatial scale, with complete coverage from latitudes 56S to 60N.<br>Data acquisition timing:<br>NOAA Coastal Lidar: 2013-2015<br>SRTM:2015<br>AW3D30: 2017<br>MERITDEM: 2018<br>GADM: 2015<br>Landscan 2010: 2015
Data exclusions	No data were excluded.
Reproducibility	Thorough QA analysis was performed to find and eliminate bugs/errors in the code. The exposure analysis system was used to integrate/compute total populations in each country, which produced accurate statistics. Inundation surfaces computed using NOAA's Coastal Lidar were also compared to NOAA's Sea Level Rise Viewer (https://coast.noaa.gov/digitalcoast/tools/slr.html) with virtually identical results.
Randomization	Not relevant
Blinding	Not relevant

Did the study involve field work? ☐ Yes ☒ No

Reporting for specific materials, systems and methods

We require information from authors about some types of materials, experimental systems and methods used in many studies. Here, indicate whether each material, system or method listed is relevant to your study. If you are not sure if a list item applies to your research, read the appropriate section before selecting a response.

Materials & experimental systems

n/a	Involved in the study	
☒	☐	Antibodies
☒	☐	Eukaryotic cell lines
☒	☐	Palaeontology
☒	☐	Animals and other organisms
☒	☐	Human research participants
☒	☐	Clinical data

Methods

n/a	Involved in the study	
☒	☐	ChIP-seq
☒	☐	Flow cytometry
☒	☐	MRI-based neuroimaging
